# Supplementary material for: A divergence between underlying and final causes of death in selected conditions: an analysis of death registries in Peru
Source: PeerJ. 2018 Nov 12;6:e5948. doi: 10.7717/peerj.5948 (PMC6237111; doi:10.7717/peerj.5948)
Supplement: Supplemental Information 1 [file peerj-06-5948-s001.docx]

# SUPPLEMENTARY MATERIAL

## Supplementary Figure 1: Flowchart of included observations in thee analysis

Total deaths in the 2015 registry

96,239

Without missing or wrong information in final cause of death

96,234

Only ≥18 years old

89,324

Missing information on habitual geographic location (i.e., foreigners), 89,225

Without cardiac arrest (ICD-10= I46) in final cause of death

81,508

Without diabetes (ICD-10=E10, E11, E13) in final cause of death

81,328

Without chronic kidney disease (ICD-10=N18) in final cause of death

80,574

Without chronic obstructive pulmonary disease (ICD-10=J41, J42, J44) in final cause of death

80,336

Without hypertension (ICD-10=I10) in final cause of death

79,597

Without selected cancers (ICD-10=C16, C61, C34, C50, C53) in final cause of death

**77,065**

Included in the analysis

## Supplementary Figure 2: Final cause of death as per the global burden of disease ICD-10 code categories in selected underlying causes in Peru, 2015


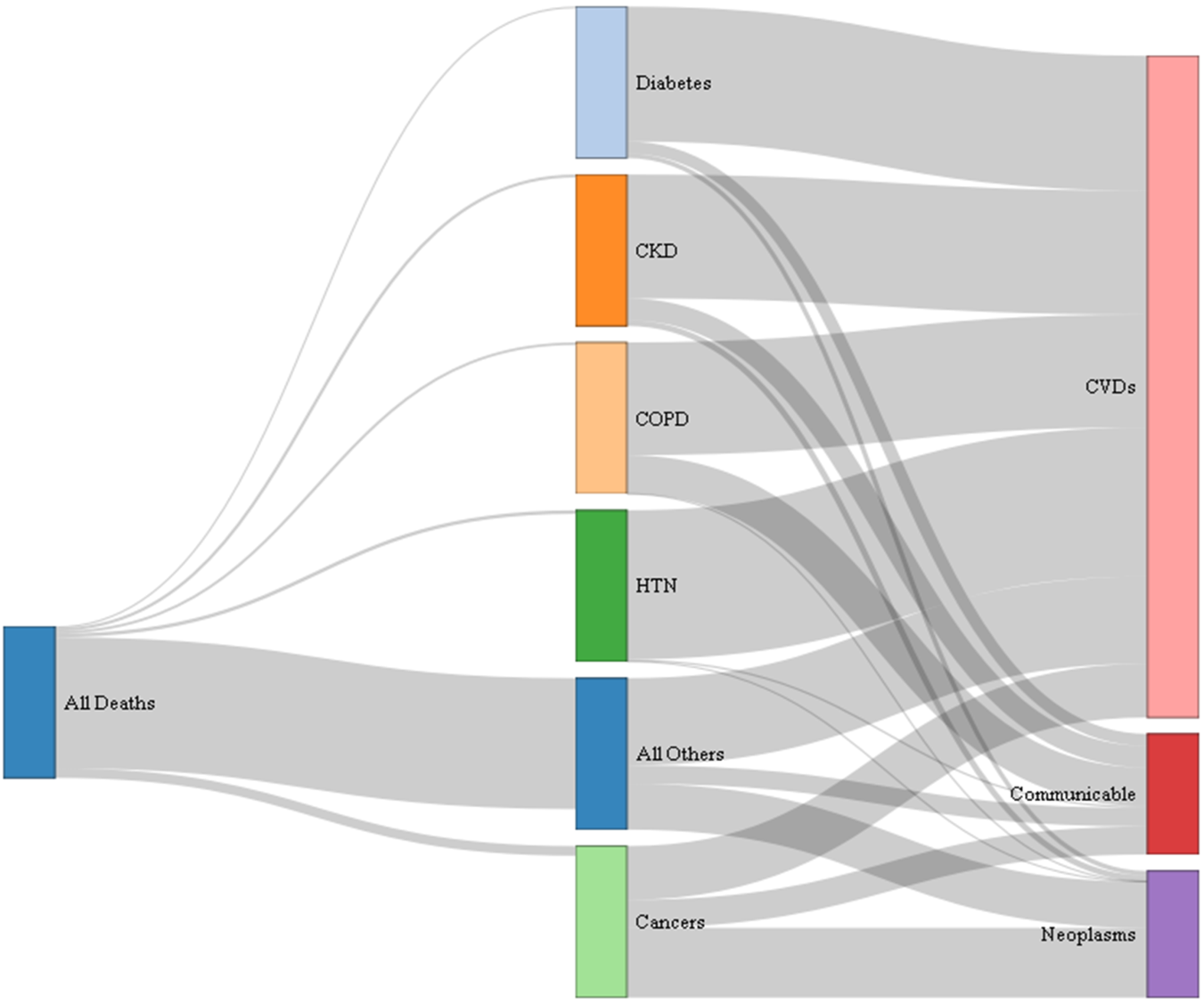


This figure is to be read horizontally: starting from all deaths, how many of these were due to the selected underlying causes of death, and of these how many were attributed to the studied final causes of death. The width of the links between nodes is relative to the proportion of deaths, i.e., the wider the link the larger the proportion. Interpretation: from all deaths that occurred in 2015, the largest proportion was due to other causes; also, when hypertension was the underlying cause of death, the largest final cause fall in the cardiovascular category. CKD = chronic kidney disease; COPD = chronic obstructive pulmonary disease; HTN = hypertension; CVD = cardiovascular diseases. Refer to Supplementary Table 6 for the exact estimates.

## Supplementary Table 1: ICD-10 codes used to define the outcome variable

| Communicable | A00-A00.9, A01.0-A14, A15-A28.9, A30-A30.9, A32-A39.9, A48.1-A48.2, A48.4-A48.5,A49.1, A50-A58, A60-A60.9, A63-A63.8, A65.0, A68-A70, A74, A74.8-A75.9, A77-A96.9, A98-A98.8, B00-B06.9, B10-B10.8, B15-B17.9, B19-B27.9, B29.4, B33-B33.1,B33.3-B33.8, B47-B48.8, B50-B53.8, B55.0, B56-B57.5, B60-B60.8, B63, B65-B67.9,B69-B72.0, B74.3-B75, B77-B77.9, B83-B83.8, B90-B92, B94.1-B94.2, B95-B95.5,B97.4-B97.6, D50.1-D50.8, D51-D52.0, D52.8-D53.9, D64.3, E00-E02, E40-E46.9, E51-E61.9, E63-E64.0, E64.2-E64.9, F07.1, G00.0-G00.8, G03-G03.8, G04-G05.8, G14-G14.6, H70-H70.9, I00, I02, I02.9, I98.0-I98.1, J01-J01.9, J02.0, J03.0, J04.0, J05.0, J09-J15.8, J16-J16.9, J20-J21.9, J36.0, K67.0-K67.8, K71.2, K71.6, K74.7-K74.8,K75.3, K76.3, K77.0, K93.0-K93.1, M03.1, M12.1, M49.0-M49.1, M73.0-M73.1, M89.6, N74.1-N74.2, N96, N98-N98.9, O00-O07.9, O09-O16.9, O20-O26.9, O28-O36.9, O40-O48.1, O60-O77.9, O80-O92.7, O96-O98.6, O98.8-P04.2, P04.5-P05.9, P07-P15.9, P19-P22.9, P23.0-P23.4, P24-P29.9, P35-P37.2, P37.5-P39.9, P50-P61.9, P70, P70.3-P72.9, P74-P78.9, P80-P81.9, P83-P84, P90-P94.9, P96, P96.3-P96.4, P96.8, R19.7, U04-U04.9,U06-U06.9, U82-U89, Z16-Z16.3 |
| --- | --- |
| Cardiovascular | B33.2, G45-G46.8, I01-I01.9, I02.0, I05-I09.9, I11-I11.9, I20-I25.9, I28-I28.8, I30-I31.1, I31.8-I41.9, I42.1-I42.8, I43-I43.9, I47-I48.9, I51.0-I51.4, I60-I63.9, I65-I66.9, I67.0-I67.3, I67.5-I67.6, I68.0-I68.2, I69.0-I69.3, I70.2-I70.8, I71-I73.9, I77-I83.9, I86-I89.0, I89.9, I98, K75.1 |
| Cancers | C00-C13.9, C15-C25.9, C30-C34.9, C37-C38.8, C40-C41.9, C43-C45.9, C47-C54.9, C56-C57.8, C58.0, C60-C63.8, C64-C67.9, C68.0-C68.8, C69-C75.8, C81-C86.6, C88-C96.9, D00.1-D00.2, D01.0-D01.3, D02.0-D02.3, D03-D06.9, D07.0-D07.2, D07.4-D07.5, D09.0, D09.2-D09.3, D09.8, D10.0-D10.7, D11-D12.9, D13.0-D13.7, D14.0-D14.3, D15-D16.9, D22-D24.9, D26.0, D27-D27.9, D28.0-D28.1, D28.7, D29.0-D29.8, D30.0-D30.8, D31-D36, D36.1-D36.7, D37.1-D37.5, D38.0-D38.5, D39.1-D39.2, D39.8, D40.0-D40.8, D41.0-D41.8, D42-D43.9, D44.0-D44.8, D45-D47.0, D47.2-D47.9, D48.0-D48.6, D49.2-D49.4, D49.6, K31.7, K62.0-K62.1, K63.5, N60-N60.9, N84.0-N84.1, N87-N87.9 |

These codes are as per the Global Burden of Disease study, which were used for sensitivity analysis. The outcome used in the primary analysis includes these codes and others as described in the methods section.

## Supplementary Table 2: Underlying causes of death by sex (p<0.001)

| **Underlying cause of death** | **Men (%)**  **[n = 41,544]** | **Women (%)**  **[n = 35,521]** |
| --- | --- | --- |
| All others | 87.46 | 85.25 |
| Diabetes | 1.01 | 1.23 |
| Chronic kidney disease | 1.98 | 1.95 |
| Chronic obstructive pulmonary disease | 1.71 | 1.77 |
| Hypertension | 2.19 | 2.54 |
| Cancers | 5.66 | 7.27 |

## Supplementary Table 3: Final cause of death by sex (p<0.001)

| **Final cause of death – sensitivity analysis outcome** | **Men (%)**  **[n = 5,452]** | **Women (%)**  **[n = 4,704]** | **Total (%)**  **[n = 10,156]** |
| --- | --- | --- | --- |
| Communicable | 13.10 | 10.16 | 11.74 |
| Cardiovascular | 61.68 | 57.61 | 59.80 |
| Neoplasms | 25.22 | 32.23 | 28.47 |
| **Final cause of death – main outcome** | **Men (%)**  **[n = 14,601]** | **Women (%)**  **[n = 13,560]** | **Total (%)**  **[n = 28,161]** |
| Communicable | 59.54 | 60.44 | 59.97 |
| Cardiovascular | 31.05 | 28.38 | 29.76 |
| Neoplasms | 9.42 | 11.18 | 10.27 |

## Supplementary Table 4: Cross-tabulation of underlying and final cause of death (p<0.001)

|  |  | **Final cause of death – Primary analysis** | | |
| --- | --- | --- | --- | --- |
| **Underlying cause of death (%)** |  | **Communicable** | **Cardiovascular** | **Neoplasms** |
|  | All others (n = 24,719) | 59.95 | 28.92 | 11.13 |
|  | Diabetes (n = 363) | 68.32 | 30.85 | 0.83 |
|  | Chronic kidney disease  (n = 511) | 79.26 | 20.16 | 0.59 |
|  | Chronic obstructive pulmonary disease (n = 382) | 86.39 | 13.61 | 0.00 |
|  | Hypertension (n = 997) | 17.55 | 82.25 | 0.20 |
|  | Cancers (n = 1,189) | 76.53 | 12.36 | 11.10 |
|  |  | **Final cause of death – Sensitivity analysis** | | |
|  |  | **Communicable** | **Cardiovascular** | **Neoplasms** |
| **Underlying cause of death (%)** | All others (n = 9,064) | 12.15 | 57.50 | 30.35 |
|  | Diabetes (n = 100) | 8.00 | 89.00 | 3.00 |
|  | Chronic kidney disease  (n = 77) | 14.29 | 81.82 | 3.90 |
|  | Chronic obstructive pulmonary disease (n = 47) | 25.53 | 74.47 | 0.00 |
|  | Hypertension (n = 581) | 1.20 | 98.45 | 0.34 |
|  | Cancers (n = 278) | 18.47 | 35.54 | 45.99 |

## Supplementary Table 5: Estimates used to plot figure 1 (Sankey plot in main manuscript)

| **Nodes 0** | **Nodes 1 [underlying causes]** | **Nodes 2 [final causes]** |
| --- | --- | --- |
| From all causes … | …to all others (84.44%) |  |
|  | …to diabetes (1.11%) |  |
|  | …to chronic kidney disease (1.96%) |  |
|  | …to chronic obstructive pulmonary disease (1.73%) |  |
|  | …to hypertension (2.35%) |  |
|  | …to cancers (6.40%) |  |
|  | From all others… | …to communicable (59.95%) |
|  |  | …to cardiovascular (28.92%) |
|  |  | …to neoplasm (11.13%) |
|  | From diabetes… | …to communicable (68.32%) |
|  |  | …to cardiovascular (29.16%) |
|  |  | …to neoplasm (0.83%) |
|  | From chronic kidney disease … | …to communicable (79.26%) |
|  |  | …to cardiovascular (20.16%) |
|  |  | …to neoplasm (0.59%) |
|  | From chronic obstructive pulmonary disease… | …to communicable (86.39%) |
|  |  | …to cardiovascular (13.61%) |
|  |  | …to neoplasm (0.00%) |
|  | From hypertension…. | …to communicable (17.55%) |
|  |  | …to cardiovascular (82.25%) |
|  |  | …to neoplasm (0.20%) |
|  | From cancers… | …to communicable (78.63%) |
|  |  | …to cardiovascular (12.36%) |
|  |  | …to neoplasm (11.10%) |

## Supplementary Table 6: Estimates used to plot supplementary figure 1 (Sankey plot in supplementary material)

| **Nodes 0** | **Nodes 1 [underlying causes]** | **Nodes 2 [final causes]** |
| --- | --- | --- |
| From all causes … | …to all others (86.44%) |  |
|  | …to diabetes (1.11%) |  |
|  | …to chronic kidney disease (1.96%) |  |
|  | …to chronic obstructive pulmonary disease (1.73%) |  |
|  | …to hypertension (2.35%) |  |
|  | …to cancers (6.40%) |  |
|  | From all others… | …to communicable (12.15%) |
|  |  | …to cardiovascular (57.50%) |
|  |  | …to neoplasm (30.35%) |
|  | From diabetes… | …to communicable (8.00%) |
|  |  | …to cardiovascular (89.00%) |
|  |  | …to neoplasm (3.00%) |
|  | From chronic kidney disease … | …to communicable (14.29%) |
|  |  | …to cardiovascular (81.82%) |
|  |  | …to neoplasm (3.90%) |
|  | From chronic obstructive pulmonary disease… | …to communicable (25.53%) |
|  |  | …to cardiovascular (74.47%) |
|  |  | …to neoplasm (0.00%) |
|  | From hypertension…. | …to communicable (1.20%) |
|  |  | …to cardiovascular (98.45%) |
|  |  | …to neoplasm (0.34%) |
|  | From cancers… | …to communicable (18.47%) |
|  |  | …to cardiovascular (35.54%) |
|  |  | …to neoplasm (45.99%) |
